# Methamphetamine Reduces Human Influenza A Virus Replication

**DOI:** 10.1371/journal.pone.0048335

**Published:** 2012-11-06

**Authors:** Yun-Hsiang Chen, Kuang-Lun Wu, Chia-Hsiang Chen

**Affiliations:** 1 Division of Mental Health and Addiction Medicine, Institute of Population Health Sciences, National Health Research Institutes, Zhunan, Taiwan; 2 Department of Psychiatry, Chang Gung Memorial Hospital at Linkou and Chang Gung University School of Medicine, Taoyuan, Taiwan; University of Regensburg, Germany

## Abstract

Methamphetamine (meth) is a highly addictive psychostimulant that is among the most widely abused illicit drugs, with an estimated over 35 million users in the world. Several lines of evidence suggest that chronic meth abuse is a major factor for increased risk of infections with human immunodeficiency virus and possibly other pathogens, due to its immunosuppressive property. Influenza A virus infections frequently cause epidemics and pandemics of respiratory diseases among human populations. However, little is known about whether meth has the ability to enhance influenza A virus replication, thus increasing severity of influenza illness in meth abusers. Herein, we investigated the effects of meth on influenza A virus replication in human lung epithelial A549 cells. The cells were exposed to meth and infected with human influenza A/WSN/33 (H1N1) virus. The viral progenies were titrated by plaque assays, and the expression of viral proteins and cellular proteins involved in interferon responses was examined by Western blotting and immunofluorescence staining. We report the first evidence that meth significantly reduces, rather than increases, virus propagation and the susceptibility to influenza infection in the human lung epithelial cell line, consistent with a decrease in viral protein synthesis. These effects were apparently not caused by meth’s effects on enhancing virus-induced interferon responses in the host cells, reducing viral biological activities, or reducing cell viability. Our results suggest that meth might not be a great risk factor for influenza A virus infection among meth abusers. Although the underlying mechanism responsible for the action of meth on attenuating virus replication requires further investigation, these findings prompt the study to examine whether other structurally similar compounds could be used as anti-influenza agents.

## Introduction

Methamphetamine (meth) is the second most widely abused drug after cannabis, and is an illicit highly-addictive stimulant for the central nervous system. Abuse of meth is a serious public health problem with more than 35 million users worldwide. Recent epidemiological studies indicated that approximately 5% of the population aged over 12 years in the United States has used meth at least once, and the rate of hospital admissions for the treatment of meth-abuse related complications has increased over three-fold than previously reported [1], [2]. Long-term abuse of meth can cause a number of negative consequences, including acute toxicity, altered behavioral and cognitive functions, and persistent neurodegenerative changes in the brain [3], [4]. Several lines of evidence have shown that meth can induce damages to dopamine terminals in the striatum and serotonin terminals in various brain regions [5]–[7].

It has been documented that meth abuse not only elicits a wide range of effects on neurons, but also decreases host resistance to pathogen infections. A growing body of evidence indicates that meth is a risk factor for human immunodeficiency virus 1 (HIV-1) infection and also for hepatitis C virus (HCV) infection [8]–[10]. The greater susceptibility to viral infection is not solely restricted to the use of contaminated injection devices, or to the high-risk sexual behavior, but also related to the deleterious effects of meth on both innate and adaptive immunity. Although the molecular basis for the action on immune suppression remains to be elucidated, meth has been shown to inhibit innate immunity in the host cells, leading to the enhancement of HIV-1 infection in human macrophages and dendritic cells, and HCV replication in human hepatic cells [11]–[13]. However, no studies have examined whether meth itself can enhance influenza A virus replication, and thus elevates influenza A virus infection and exacerbates influenza illness in meth abusers.

Human influenza A viruses are enveloped and contain eight different strands of single-stranded negative-sense RNA associated with nucleoprotein and RNA polymerase, which encode 11 viral proteins [14]. The viral infection and replication mainly occur in the ciliated columnar epithelial cells of the upper respiratory tract [15], [16]. Influenza A virus infection is a common cause of respiratory illness in humans, and the epidemics occur almost annually in many countries with attack rates of over ten percent of the population, in spite of the wide availability of influenza vaccines [17], [18]. The persistent threat of currently circulating human influenza A viruses (H1N1, H1N2, and H3N2), and the recent outbreaks of avian influenza A virus (H5N1) and swine-origin influenza A virus (H1N1) have raised serious concerns about the potential of a new influenza pandemic [19]–[22].

The present study was undertaken to investigate the effects of meth on influenza A virus replication in human lung epithelial cells, and also to explore the underlying mechanism involved in the action of meth on this virus. Our data demonstrate that meth reduces influenza A virus replication and spread *in vitro* without enhancing anti-viral interferon responses, and encourage further studies to investigate whether other structurally similar compounds can be used as antiviral drugs against influenza A virus.

## Materials and Methods

### Chemicals

Meth was obtained as a powder format from National Bureau of Controlled Drugs, Department of Health, Taiwan. As a stock, meth was dissolved in phosphate-buffered saline (PBS) at a concentration of 250 mM, sterilized by filtering through membrane filters with a pore size of 0.2 µm, and stored at −20°C until use. The stock of chloroquine diphosphate salt (C6628, Sigma) was prepared as described above at a concentration of 20 mM.

### Cell Lines and Virus

Human lung epithelial A549 cells (BCRC-60074, Bioresource Collection and Research Center, Taiwan) and Madin-Darby canine kidney (MDCK) cells were grown as monolayers in Dulbecco’s modified eagle’s medium (DMEM) supplemented with 10% (v/v) heat-inactivated fetal bovine serum (FBS), antibiotics (100 U/ml penicillin, 100 µg/ml streptomycin), and a nonessential amino acid mixture (0.1 mM). Both cell lines were maintained at 37°C in a humidified incubator with 5% CO_2_. The human influenza A/WSN/33 (H1N1) wild-type virus and MDCK cell line [23], used in this study, were kindly provided by Prof. Richard E. Randall (University of St Andrews, UK). To propagate the influenza virus, confluent MDCK cells were inoculated with the virus at a multiplicity of infection (MOI) of 0.001 PFU/cell in serum-free DMEM containing N-acetyl trypsin (2 µg/ml; T6763, Sigma) at 37°C [24]. The culture medium was collected at 48 h post-infection, cell debris was removed by centrifugation at 1,000 ×g for 5 min, and virus titers were determined by plaque assay on MDCK cells. Unless otherwise indicated, all reagents were purchased from Invitrogen Corporation.

### Plaque Assay

Confluent MDCK cells grown in six-well plates were washed once with Dulbecco’s phosphate-buffered saline (DPBS) and then inoculated with 10-fold serially diluted viral suspensions in 1 ml of DMEM at 37°C, with gently rocking for 1 h. After replacing the inoculation medium with 2 ml of the warm overlay medium [DMEM supplemented with 1% (w/v) agarose (50080, Lonza) and 2 µg/ml N-acetyl trypsin] for each well, plates were left at room temperature for 30 min to solidify the overlay medium, and cells were then incubated at 37°C for 3 days, with plates inverted. To visualize the plaques, cells were fixed with 4% (v/v) formaldehyde (33220, Sigma) in PBS for 2 h and then incubated with staining buffer [composed of 0.05% (w/v) crystal violet (C3886, Sigma), 20% (v/v) ethanol, and 1% (v/v) methanol in distilled water] at room temperature for 30 min, followed by a brief wash in running tap water. Plaques were photographed and counted; and the virus titer was expressed as plaque formation units per milliliter (PFU/ml).

The anti-influenza virus activity of meth was determined by plaque-reduction assay in A549 cells. Briefly, confluent A549 cells grown in 6-well plates were treated with meth at various concentrations (2.5, 25, 125, 250 µM) for 24 h, and then subjected to the treatment as described in the plaque assay, except for that meth was added in the virus inoculation medium and overlay medium at indicated concentrations.

### Viral Growth Analysis

A549 cells were seeded (5×10^4^ cells/cm^2^) in 25T flasks 24 h before being treated with meth at various concentrations (2.5, 25, 125, 250 µM) for 24 h at 37°C. The concentrations of meth used here are similar to the blood levels found in meth abusers [12], [25], [26]. Cells were washed twice with DPBS and then infected with influenza A/WSN/33 (H1N1) virus at an MOI of 0.001 PFU/cell in serum-free DMEM containing N-acetyl trypsin (2 µg/ml) and meth at respective concentrations. The culture media were collected at various time points post-infection (24, 30, 48 h), and stored at −80°C until being subjected to determine virus titers by plaque assays as described above.

### Time-course Assay

A549 cells were seeded (5×10^4^ cells/cm^2^) in 6-well plates and grown overnight before being treated with meth (at 250 µM) or left untreated for 24 h at 37°C. After that, cells were infected with influenza A/WSN/33 virus at an MOI of 1 PFU/cell in serum-free DMEM without the presence of trypsin (single-cycle growth). Meth was added to or removed from the culture medium at different time periods: pre-adsorption (−24∼0 h), adsorption (0∼1 h), early post-infection (1∼4 h), and late post-infection (4∼ 22 h). Cells were washed twice with DPBS between each incubation period. The culture media were collected at 22 h post-infection, and stored at −80°C until being subjected to determine virus titers by plaque assays as described above.

**Figure 1:**
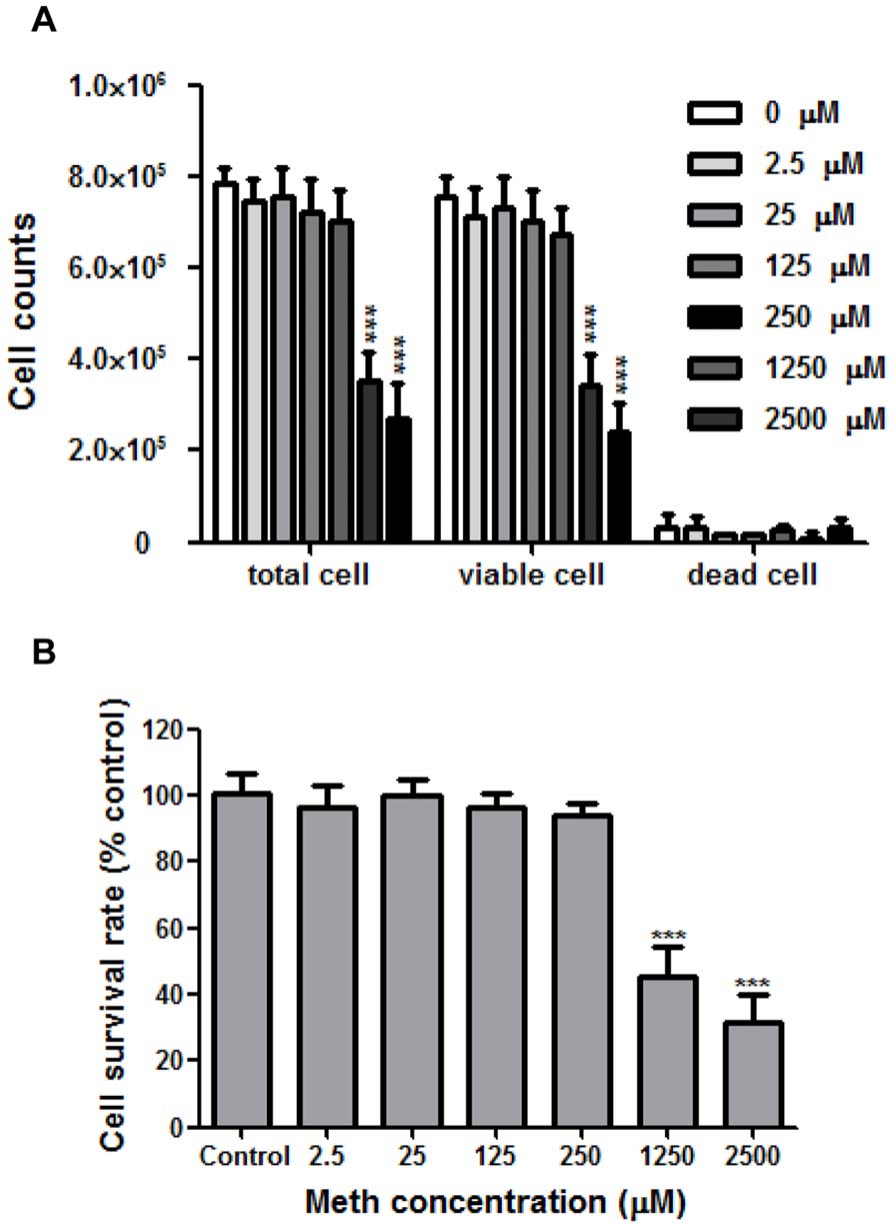
The cytotoxic effect of meth on human lung epithelial A549 cells. A549 cells were treated with meth at indicated concentrations at 37°C for 72 h. (A) The trypan blue dye exclusion assay was performed to count the total, viable, and dead cells. (B) The cell survival rate was calculated, and data were expressed as percentages of viable counts in meth-treated groups relative to that in the meth-untreated group (control). The results are means ± SD of five replicates from a representative result of three independent experiments. Significant differences were indicated (***: p < 0.0001 versus control).

### Immunofluorescence Staining

The following procedures were performed at room temperature, unless otherwise stated. At 24 h post-infection, human epithelial A549 cells (grown on 12-mm glass coverslips) were washed twice with PBS, fixed with 4% formaldehyde in PBS for 10 min, and permeabilized with 0.3% Triton X-100 in PBS for 10 min. The cells were incubated with 4% bovine serum albumin (BSA; 10857, USB Corp.) in PBS to block nonspecific binding of the antibodies, and stained with a mouse monoclonal antibody against the nucleoprotein (NP) of human influenza A virus (1:1000; sc-101352, Santa Cruz) for 1 h. After washing with PBS three times for 5 min each, cells were incubated with an Alexa-Fluor-488-conjugated goat polyclonal antibody against mouse IgG (1:1,000; A11029, Invitrogen) for 30 min, followed by washing with PBS three times for 5 min each. The cells were fixed again with 4% formaldehyde for 10 min, and washed with distilled water once. After that, the coverslips were mounted on glass slides by using a mounting medium (H-1200, Vector Laboratories), which contains 4′, 6-diamidino-2-phenylindole (DAPI; 1.5 µg/ml) for localizing the nucleus; then, cells were examined and fluorescence images were obtained by using the Axiovert 200 M microscope system (Zeiss).

**Figure 2:**
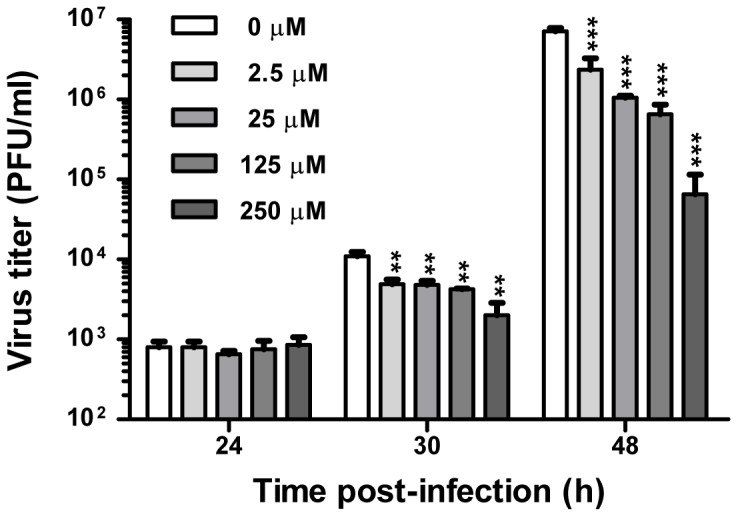
Meth reduces influenza A virus propagation in human lung epithelial A549 cells. A549 cells were left untreated or treated with meth at indicated concentrations for 24 h, followed by infection with human influenza virus strain A/WSN/33 (H1N1) at an MOI of 0.001 PFU/cell (trypsin present) in the presence of meth at respective concentrations. Virus progenies were collected at indicated time points, and subjected to plaque assays in MDCK cells to determine virus titers. Values are means ± SD of three replicates from a representative result of three independent experiments. Significant differences from the meth-untreated control were indicated (**: p < 0.01, ***: p < 0.0001).

### Trypan Blue Dye Exclusion Assay

The effect of meth treatment on the viability of human lung epithelial A549 cells was determined by the trypan blue dye exclusion assay. A549 cells were seeded (1×10^4^ cells/cm^2^) in 6-well plates and grown at 37°C for 24 h, followed by the replacement of culture medium with fresh medium supplemented without (as control) or with meth at indicated concentrations (2.5, 25, 125, 250, 1250, 2500 µM). After incubation at 37°C for 24 h, cells were re-treated without or with meth as described above for 48 h; after that, cells were harvested by trypsinization and stained with 0.4% trypan blue dye (T8154, Sigma). The positively- and negatively-stained cells were counted as dead and viable cells, respectively, by using a haemocytometer under a light microscope. The cell survival rate was calculated by the formula: [(the total number of viable counts in the meth-treated group)/(the total number of viable counts in the control group)]×100%.

**Figure 3:**
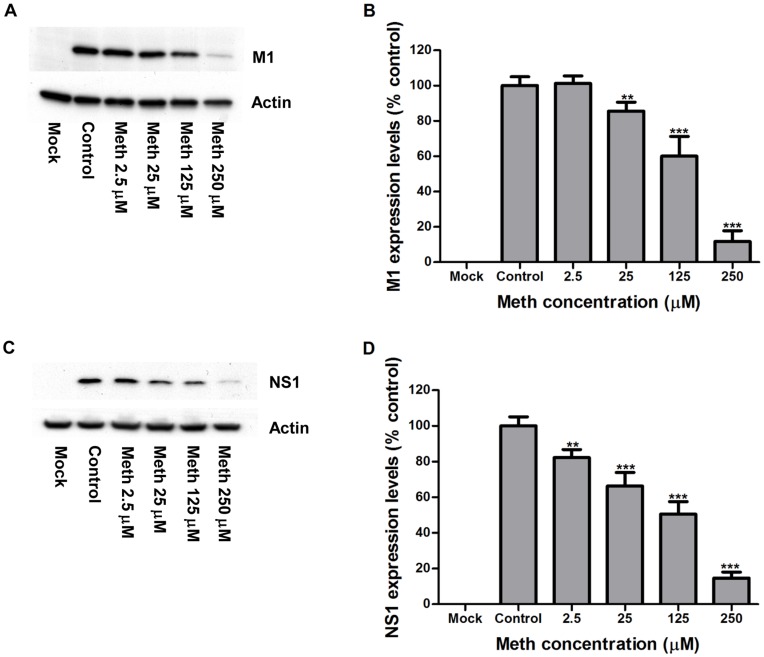
Meth reduces synthesis of influenza viral proteins in human lung epithelial A549 cells. A549 cells were left untreated (control) or treated with meth at indicated concentrations for 24 h, and then infected with human influenza virus strain A/WSN/33 (H1N1) at an MOI of 0.001 PFU/cell in the presence of trypsin (multi-cycle growth) and meth at respective concentrations. Whole cell lysates were prepared at 48 h post-infection and subjected to Western blot analysis using antibodies against viral matrix protein-1 [M1] (A), viral nonstructural protein-1 [NS1] (C), and cellular actin. Mock: meth-untreated cells without influenza infection. A representative result from three independent experiments is shown. The expression levels of detected proteins were measured by densitometric analysis. M1 (B), and NS1 (D) levels were normalized by actin levels, and the relative optical density values were expressed as percentages of control. The results represent mean values ± SD of three replicates from a representative result of three independent experiments. Significant differences from the control were indicated (**: p < 0.01, ***: p < 0.0001).

**Figure 4:**
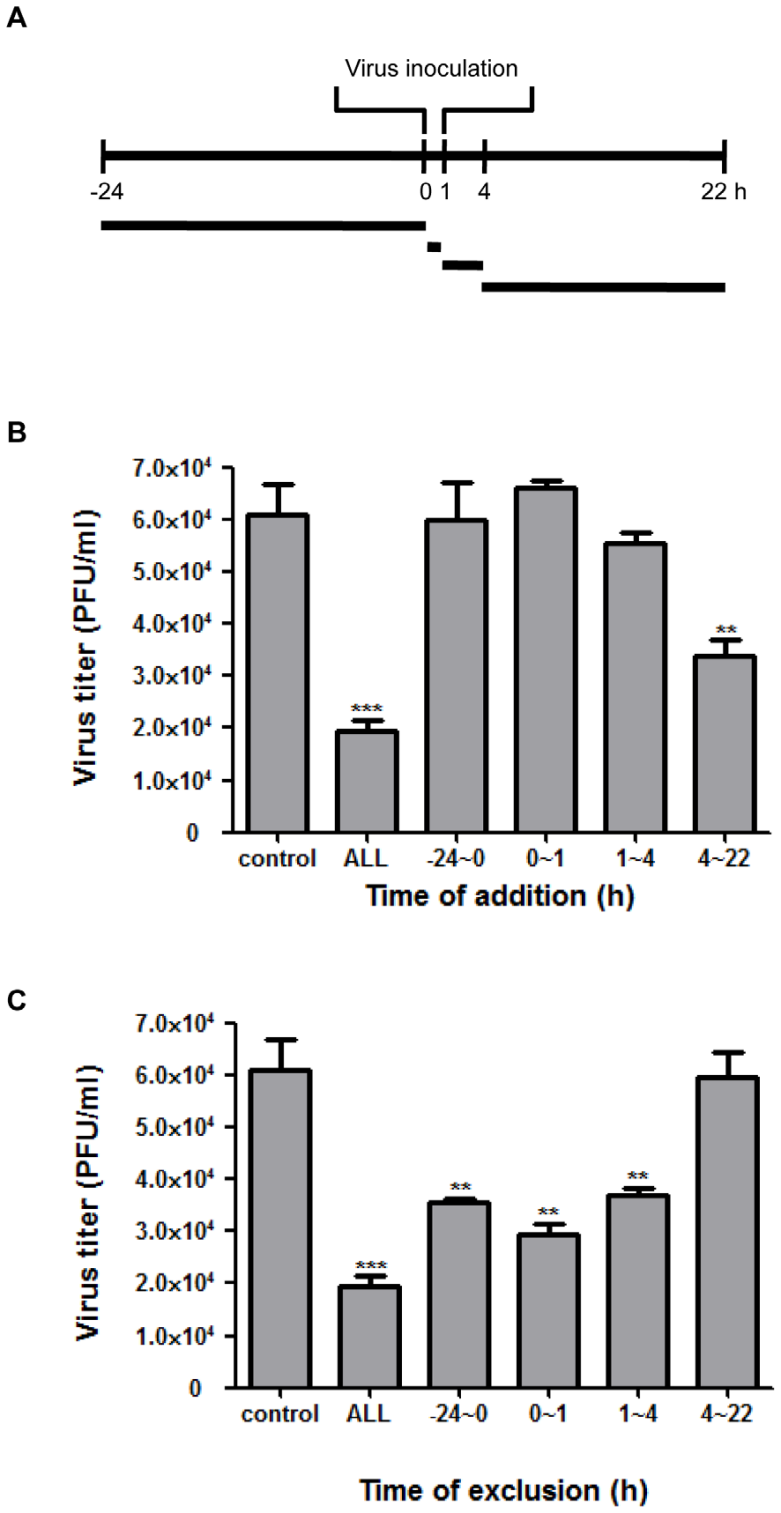
Meth reduces influenza A virus propagation at specific steps of the virus replication cycle. The assay to determine the effectiveness of meth in reducing influenza A virus replication with regard to the time-course of meth-exposure was performed in A549 cells as described in Materials and Methods. Cells were exposed to meth at 250 µM or left unexposed as the control (meth was not present in all incubation periods), followed by infection with influenza A/WSN/33 (H1N1) virus at an MOI of 1 PFU/cell in the absence of trypsin (single-cycle growth). Meth was added to (time-of-addition) or removed from (time-of-exclusion) the culture medium at different incubation periods as illustrated in the diagram (A). Virus progenies were collected at 22 h post-infection and subjected to plaque assays in MDCK cells to determine virus titers (B, C). ALL: meth was present in all incubation periods (−24∼22 h). The virus titer is expressed as plaque formation units per milliliter (PFU/ml). The results represent mean values ± SD of three replicates from a representative result of three independent experiments. Significant differences from the control were indicated (**: p < 0.01, ***: p < 0.0001).

### Western Blotting

Unless otherwise indicated, the following procedures were performed at room temperature. The cells were washed twice with PBS, and lysed in 2× Laemmli buffer (containing 4% SDS, 125 mM Tris-HCl pH6.8, 10% β-mercaptoethanol, 20% glycerol, and 0.004% bromophenol blue in distilled water). Whole cell lysates were passed through a 25-gauge needle several times to reduce the viscosity, heated at 90°C for 10 min, and then separated by electrophoresis on 12% SDS-polyacryamide gels. The separated proteins were electrophoretically transfered to PVDF membranes (RPN303F, GE Healthcare), and then subjected to immunoblotting by using appropriate antibodies. The membranes were incubated with blocking buffer [containing 5% skim milk (70166, Sigma), and 0.1% Tween-20 in PBS] for 1 h, and then incubated with the target-specific primary antibody at an appropriate dilution in 10 ml blocking buffer for 2 h, with gentle agitation. After washing with 0.1% Tween-20 (in PBS) three times for 10 min each, the membranes were incubated with the secondary antibody (specific to the immunoglobulin isotype of the primary antibody) at an appropriate dilution in 10 ml blocking buffer for 1 h, followed by the washing procedure as described above. Note, for the primary antibody specific to tyr-701 phospho-STAT1, the skim milk content of blocking buffer was replaced with BSA, and the incubation with primary antibody was performed at 4°C overnight. The target proteins on the membrane were detected by using an enhanced chemiluminescence reagent (RPN2106, GE Healthcare), and the intensity of generated images on X-film (NEF596, Kodak) was quantified by ImageQuant TL software (GE Healthcare).

**Figure 5:**
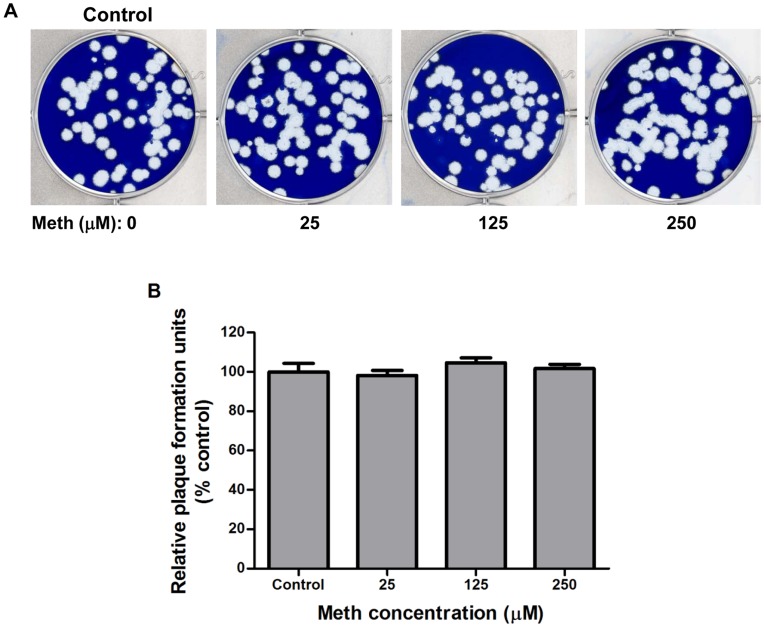
Meth does not directly reduce the biological activities of human influenza virus strain A/WSN/33 (H1N1). Influenza A viruses were incubated with meth at indicated concentrations in serum-free culture medium at 37°C for 24 h, and subjected to plaque assay in MDCK cells. (A) Plaque phenotype. A representative result of three independent experiments with similar results is shown. No obvious differences of plaque size were observed between groups. (B) Relative plaque numbers. The results are means ± SD of three replicates and expressed as percentages of plaque numbers of meth-pretreated viruses relative to that of meth-untreated viruses. No significant differences in plaque numbers were shown between groups.

Three mouse monoclonal antibodies against cellular actin (1:20,000; MAB1501, Millipore), influenza A virus NS1 (1:2,000; sc-130568, Santa Cruz), and influenza A virus M1 proteins (1:2,000; GTX76107, clone GA2B, GeneTex), two rabbit monoclonal antibodies against STAT1 (1: 2,000; EPYR2154, Epitomics), and tyr-701phospho-STAT1 (1:2,000; 9167, clone 58D6, Cell Signaling), and one rabbit polyclonal antibody against cellular MxA protein (1:4000; GTX110256, GeneTex) were used as primary antibodies. Two horseradish peroxidase-conjugated goat polyclonal antibodies against mouse IgG (GTX213111-01, GeneTex) and rabbit IgG (GTX213110-01, GeneTex) were used as secondary antibodies.

**Figure 6:**
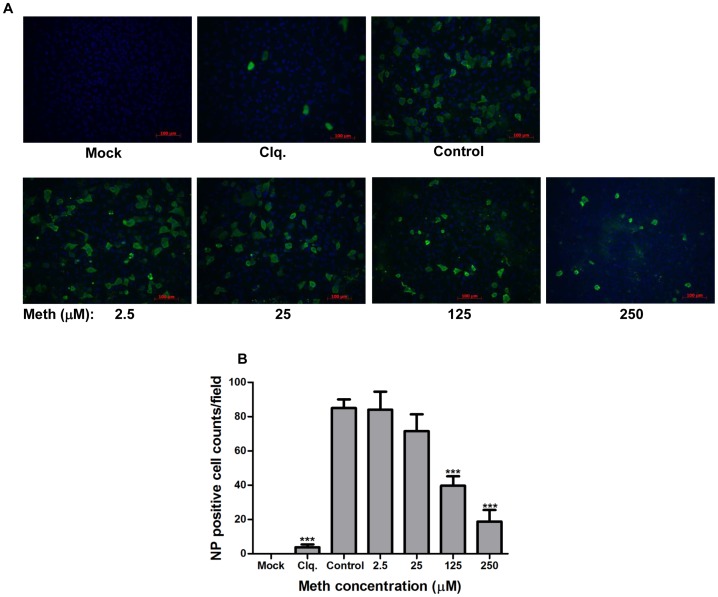
Meth reduces susceptibility to influenza A virus infections in human lung epithelial A549 cells. (A) A549 cells grown on glass coverslips were left un-treated, or treated with chloroquine (Clq.; 10 µM; as positive control) or meth at indicated concentrations, followed by the infection with influenza A/WSN/33 (H1N1) virus at an MOI of 1 PFU/cell in the absence of trypsin (single-cycle growth) and presence of the corresponding drugs at indicated concentrations. At 24 h post-infection, cells were fixed with formaldehyde and subjected to immunofluorenscence staining for detecting the infected cells by using an antibody against viral nucleoprotein (NP; green); cellular nuclei were located by DAPI staining (blue). A representative result from three independent experiments is shown. Mock: cells were neither exposed to the drugs nor infected with the virus. Scale bar: 100 µm. (B) NP-positive cells were counted from ten microscopic fields with >90% cell confluence. Data are expressed as mean values ± SD from a representative result of three independent experiments. Significant differences from the drug-untreated infected group (control) were indicated (***: p < 0.0001).

### Statistical Analysis

All results were expressed as means ± standard deviation (SD), and analyzed using GraphPad Prism 5.0 statistical program. To evaluate the significance of difference between groups, statistical analysis was performed by one-way analysis of variance (ANOVA) followed by Bonferroni’s multiple comparison test as appropriate. Statistical significance is considered when the p value is < 0.05.

**Figure 7:**
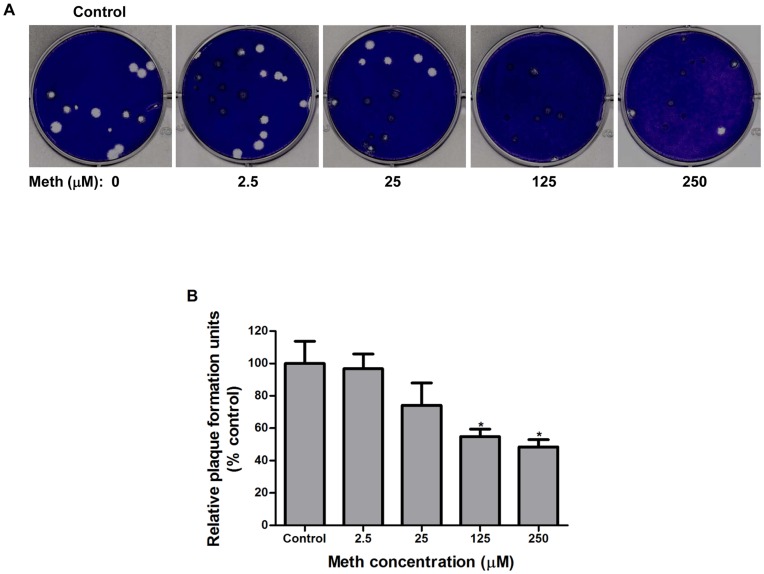
Meth reduces plaque formation in human lung epithelial A549 cells infected with influenza A viruses. The plaque-reduction assay was performed in A549 cells as described in Materials and Methods. Cells were exposed to meth at indicated concentrations or left unexposed as the control, followed by infection with influenza A/WSN/33 (H1N1) virus in the presence of meth at indicated concentrations. (A) Plaque phenotype. A representative result of three independent experiments with similar results is shown. The size of plaques under conditions of meth treatments at 125 and 250 µM appeared to be smaller than that in the condition without meth treatment. (B) Relative plaque numbers. The results are means ± SD of three replicates, and expressed as relative ratios of plaque numbers in meth-treated groups to that in the meth-untreated group (control). Significant differences from the control were indicated (*: p < 0.05).

## Results

### Meth Used at Pharmacological Concentrations is Not Cytotoxic to Human Lung Epithelial A549 Cells

To investigate whether meth exerts cytotoxic effects on human lung epithelial A549 cells, we treated the cells with meth for 72 h at different concentrations (2.5, 25, 125, 250, 1250, 2500 µM), or left the cells un-treated, and measured their viable rate by trypan blue dye exclusion assays. The meth levels ranging 2.5∼250 are comparable to that found in the blood of meth abusers [12], [25], [26]. As shown in Fig. 1, the viable cell counts were not significantly different between the un-treated group and the groups treated with meth at 2.5∼250 µM, but a notable reduction was observed in the groups treated with meth at high concentrations (1250∼2500 µM); a similar result was also found for the comparison of total cell counts. The dead cell counts were similar between the un-treated group and the groups treated with meth at 2.5∼2500 µM. In addition, there were no significant differences in the cell survival rate between cells without exposure to meth and cells exposed to meth at 2.5∼250 µM; however, when cells were exposed to meth at 1250 and 2500 µM, the cell survival rates were dramatically reduced by 50% and 70%, receptively. Taken together, these results demonstrate that meth has no apparent cytotoxic effects on A549 cells at the pharmacological concentration range, but can significantly inhibit cell proliferation at high concentrations in a dose dependent manner.

### Meth Reduces Influenza A Virus Replication in Human Lung Epithelial A549 Cells

To examine whether meth has any effects on influenza A virus replication *in vitro*, A549 cells were grown for 24 h in the medium supplemented with meth at a concentration range (2.5∼250 µM) without cytotoxic effects, followed by the infection with influenza A/WSN/33 (H1N1) virus at an MOI of 0.001 PFU/cell in the presence of meth at respective concentrations. The progeny viruses were collected at different time points and titrated by plaque assays. At 24 h post-infection, there were no obvious differences in the virus titer between meth-treated and meth-untreated (control) groups. At 30 and 48 h post-infections, the virus titers were significantly lower in the meth treated groups than that in the control, and this reduction is in a dose-dependent manner; especially, at 48 h post-infection, the virus grew to titers of ∼3-, 7-, 10-, and 100-fold lower at meth concentrations of 2.5, 25, 125, and 250 µM, respectively (Fig. 2). In addition, at 48 h post-infection, the whole cell lysates were examined by Western blotting, and the expression levels of NS1 and M1 viral proteins were significantly reduced in the meth-treated groups in a dose-dependent manner at a concentration range of 2.5∼250 µM (Fig. 3). The effect of meth on influenza protein synthesis closely reflects the effect of meth on influenza replication in A549 cells. Taken together, these results demonstrate that meth, used at a pharmacological concentration range, can attenuate rather than enhance influenza A/WSN/33 (H1N1) virus propagation *in vitro*.

**Figure 8:**
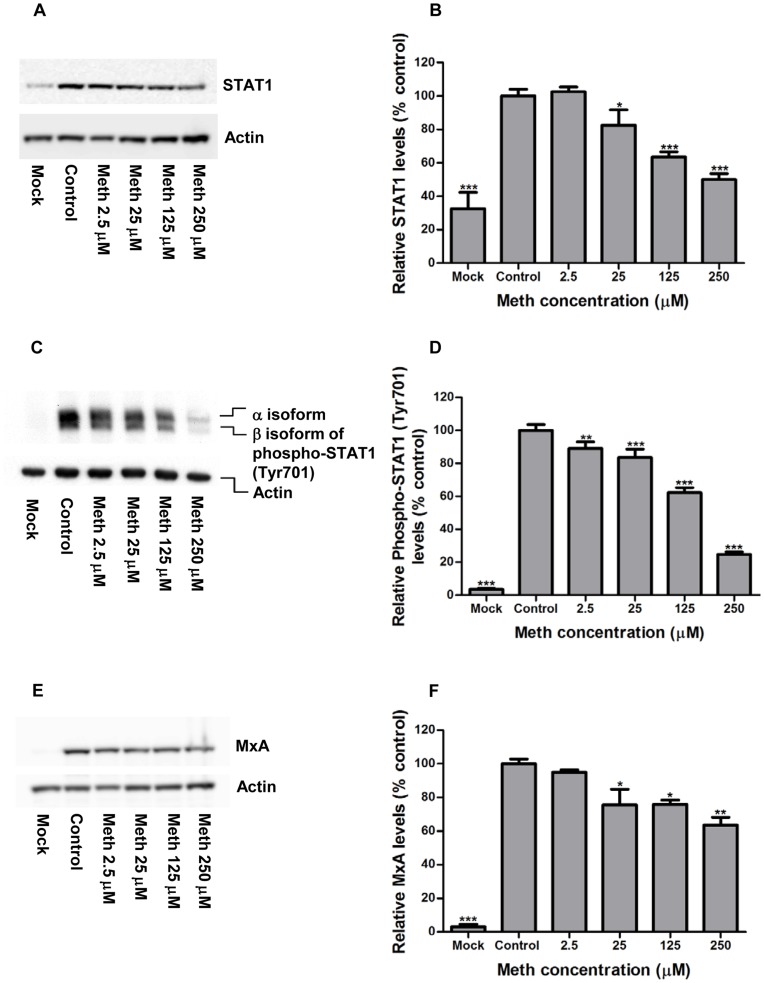
Effects of meth on influenza infection-induced IFN responses in human lung epithelial A549 cells. A549 cells were left untreated (control) or treated with meth at indicated concentrations for 24 h, followed by infection with human influenza virus strain A/WSN/33 (H1N1) at an MOI of 0.001 PFU/cell in the presence of trypsin (multi-cycle growth) and meth at respective concentrations. At 48 h post-infection, whole cell lysates were prepared and subjected to Western blot analysis using antibodies against cellular STAT1 (A), phospho-STAT1 [Tyr701] (C), MxA (E), and actin. Mock: meth-untreated cells without influenza infection. A representative result from three independent experiments is shown. The levels of detected proteins were measured by densitometric analysis. The expression levels of STAT1 (B), phospho-STAT1 [Tyr701] (D), and MxA (F) were normalized by actin levels, and the relative optical density values are expressed as percentage of control. The results represent mean values ± SD of three replicates from a representative result of three independent experiments. Significant differences from the control were indicated (*: p < 0.05, **: p < 0.01, ***: p < 0.0001).

### Meth Exerts an Anti-influenza Effect Predominantly during the Viral Replication Stage after Virus Infection

In order to investigate which parts of the virus replication cycle are affected by meth, we determine the incubation time periods of meth needed to reduce virus production from infected A549 cells. Meth was added to or excluded from the culture medium at different time periods during the course of single-cycle infection. The supernatants from infected cells were harvested at 22 h post-infection for plaque assay to determine virus yields in each meth-exposure condition. As shown in Fig. 4, when meth was added in all incubation periods (−24∼22 h), the virus production was significantly reduced, compared with that in the group without any meth-exposure. In the time-of-addition assay (Fig. 4B), an inhibitory effect of meth on virus production was observed when meth was present during the late post-infection (4∼22 h), but not pre-adsorption (−24∼0 h), adsorption (0∼1 h), or early post-infection period (1∼4 h). Furthermore, in the time-of-exclusion assay (Fig. 4C), a significant reduction of virus yields was found when meth was excluded from the medium during the pre-adsorption, adsorption, or early post-infection period, but no significant reduction was shown when meth was excluded during the late post-infection period. Virus production in the medium excluded before the late post-infection period was not detected at least under our assay conditions (data not shown). Taken together, these results indicate that meth might primarily target the viral replication stage rather than the entry or adsorption stage in the infected cells, resulting in the reduction of virus production.

### Meth has No Direct Inhibitory Effects on the Biological Activities of Influenza A Virus Particles

To investigate whether meth can affect the biological activities of virus particles to attenuate the virus production in meth-treated host cells, we pre-incubated the influenza A/WSN/33 (H1N1) virus stock with serum-free DMEM supplemented with meth at different concentrations (2.5, 25, 125, 250 µM) at 37°C for 24 h, followed by the conduction of plaque assays. There were no significant changes in the virus titer and plaque size between viruses without exposure to meth and viruses exposed to meth at different concentrations 2.5∼250 µM (Fig. 5), indicating that meth at this concentration range causes no direct damage to influenza A virus particles to attenuate their replication in the host cells.

### Meth Reduces Susceptibility to Influenza Infections in Human Lung Epithelial A549 Cells

We further investigated whether the reduced replication of influenza in meth-exposed cells could be caused by reducing susceptibility to influenza infections in the host cells. A549 cells grown on glass coverslips were left un-treated or treated with meth (2.5∼250 µM) or chloroquine (10 µM; as positive control), followed by the infection with influenza A/WSN/33 (H1N1) virus at an MOI of 1 PFU/cell in the absence of trypsin (single-cycle growth) and presence of the corresponding drugs at respective concentrations. At 24 h post-infection, cells were fixed with formaldehyde and stained with an antibody specific to the viral nucleoprotein (NP), and the number of influenza-infected cells was examined by fluorescence microscopy. As shown in Fig. 6, exposure to meth reduced the numbers of influenza-infected cells in a dose depend manner (25∼250 µM) with statistical significance at concentrations of 125 and 250 µM. When the anti-influenza virus activity of meth was examined by plaque-reduction assays (Fig. 7), the numbers of virus plaques were also reduced with statistical significance at concentrations of 125 and 250 µM. In addition, the size of virus plaques in the presence of meth at concentrations of 125 and 250 µM appeared to be smaller than that in the absence of meth exposure. Taken together, these results demonstrate that exposure to meth reduces susceptibility to influenza infections and attenuates virus spread in human lung epithelial A549 cells.

### Effects of Meth on Influenza Infection-induced IFN Responses in Human Lung Epithelial A549 Cells

The type Ι interferon (IFN) response is critical in antiviral defenses against all kinds of viruses. In response to virus infection, infected cells synthesis and secret type Ι IFNs, which activate infected cells and nearby cells to produce a broad range of antiviral proteins, leading to the inhibition of further viral growth and spread [27], [28]. Since the expression level and phosphorylation of the transcription factor STAT1 play a critical role in the regulation of type I IFN signaling, and myxovirus resistance protein (MxA) is an important IFN-inducible gene product in fighting influenza infections [29], we intend to know whether the reduction of influenza replication in meth-exposed cells is related to the up-regulation of these antiviral mediators. To test this, A549 cells were treated with meth at various concentrations for 24 h, followed by infection with human influenza virus strain A/WSN/33 (H1N1) at an MOI of 0.001 PFU/cell in the presence of trypsin (multi-cycle growth) and meth. Whole cell lysates were prepared at 48 h post-infection and subjected to Western blot analysis using antibodies against STAT1, phospho-STAT1 (Tyr701), MxA, and actin. As shown in Fig. 8, influenza infection enhanced the expression levels of STAT1, phospho-STAT1, and MxA in A549 cells in the presence or absence of meth. When compared with the control group (infected cells without meth exposure), the influenza infection-induced levels of these three antiviral mediators were reduced rather than increased in the meth-treated groups in a dose dependent manner. Taken together, these results suggest that meth reduces influenza virus replications without enhancing infection-induced IFN responses in A549 cells.

## Discussion

Meth is a widely abused and extremely addictive psychostimulant, which affects not only the central nervous system but also the immune system. Previous studies have demonstrated that meth is able to enhance infection and replication of HIV-1 in macrophages and dendritic cells, and HCV in hepatocytes [11]–[13]. In the present study, we show that meth used at pharmacologically relevant levels suppresses rather than enhances influenza A virus replication in human lung epithelial cells, which is consistent with the reduced susceptibility to influenza infection and synthesis of viral proteins. The suppression of viral replication is not due to inhibition of viral biological activities, reduction of cellular survival, or enhancement of infection-induced IFN responses. Although further *in vivo* investigation is needed, our results suggest that meth might not enhance influenza A virus infection and spread among meth abusers. In addition, elucidation of the mechanism(s) responsible for meth’s action on influenza A virus replication may help to devise novel strategies against influenza A virus infection in all populations.

In response to virus infections, infected cells synthesis and secret type Ι IFNs (including IFN-α and IFN-β), which control viral infections by sending signals to the nucleus through JAK-STAT pathway to activate the expression of various antiviral proteins (such as MxA) [28]. In fact, our data demonstrated that the reduced replication of influenza virus in the presence of meth was likely not caused by the enhancement of infection-induced IFN responses, since the levels of STAT1, phospho-STAT1, and MxA closely correlated with the levels of influenza virus replication. This study suggests that meth may reduce the susceptibility to influenza virus infections through IFN-independent mechanisms. Of note, however, the present study only examines meth’s effect on influenza infections in epithelial cells *in vitro*, but whether meth’s action on immune suppression can affect the susceptibility to influenza infections *in vivo* requires additional studies.

Apart from IFN-dependent antiviral responses, the cellular redox status has also been found to be important in regulating viral replication and infectivity [30], [31]. Several studies have shown that antioxidants can attenuate influenza virus replication *in vitro* and *in vivo*, and accumulating evidence has suggested that a more oxidized environment would favor influenza virus replication [32]–[35]. Although meth can cause destruction of dopaminergic terminals by inducing oxidative stress in the brain, chronic administration of meth has been shown to up-regulate antioxidant enzymes (such as superoxide dismutase) and augment the antioxidant activity in the plasma [36]. It remains to be investigated whether meth can induce an up-regulation of the antioxidant system to reduce the susceptibility to influenza infections in lung epithelial cells.

Recently, the anti-malaria drug, chloroquine, was suggested to be used as an agent against influenza virus infections, largely based on its biochemical property [37], [38]. As an acidic organelle tropic weak base, chloroquine increases the pH of endosomal, lysosomal and trans-Golgi network vesicles leading to dysfunction of proteases and several enzymes involved in the post-translational modification process. The rise of pH in acidic vesicles inhibits influenza virus replication through reducing the efficiency of virus un-coating in endosomal compartments, and inhibiting the post-translational modification of viral envelope glycoproteins within the Golgi apparatus [39]. Meth and its metabolite, amphetamine, have been found to act as a lipophilic weak base, like chloroquine, to increase the pH of intracellular organelles in several cell types, including neurons, macrophages, and dendritic cells [26], [40], [41]. Thus, the alkalizing effect of meth on cellular acidic organelles might provide a possible explanation for the reduction of influenza A virus replication in lung epithelial cells exposed to meth, but further experimental evidence is definitely needed to support this speculation.

In fact, meth has been demonstrated to enhance HCV replication in hepatocytes in association with compromising IFN-alpha mediated innate immunity, and HIV replication in dendritic cells and macrophages in association with up-regulating virus entry coreceptors [11]–[13]. However, we observed that meth attenuates influenza A virus replication in lung epithelial cells without enhancing infection-induced IFN responses, while the responsible mechanism(s) remains unknown. The discrepancy of meth’s action on virus replication could partly be due to differences in cell types used and/or vulnerability of virus replication cycles, although further investigation is needed.

Influenza virus infections are frequently associated with epidemics and pandemics of respiratory diseases, adversely affecting the health and economies of global populations. Although influenza virus vaccines have been effective in controlling infections [42], the gradual antigenic change of viral surface antigens, resulting from spontaneous point mutations and genetic reassortment (exchanging genetic segments between two or more virus strains), has complicated the vaccine composition and necessitated the annually administration of seasonal influenza vaccines [43], [44]. Therefore, antiviral drugs play an important role in the treatment and prevention of influenza virus infections.

Currently, two classes of antiviral drugs, adamantanes and neuraminidase inhibitors, are approved for use against influenza virus infections [45]. Although adamantanes have been used for decades, the rapid emergence of drug resistance, and their central nervous system side effects have severely diminished their usefulness for the treatment and prophylaxis of influenza virus infections [46], [47]. While resistance to neuraminidase inhibitors has been relatively infrequently reported, compared with resistance to adamantanes, an increasing emergence of resistance to oseltamivir (a neuraminidase inhibitor) has been a cause for concern [48], [49]. Thus, a demand for effective anti-influenza agents is particularly important and urgent, without doubt.

The present work has demonstrated that meth suppresses human influenza A virus replication in human lung epithelial cells. This finding strongly encourages future work to investigate whether other compounds, structurally similar to meth, can inhibit influenza A virus production and be used to prevent or alleviate influenza A virus infection. For instance, several compounds, such as ephedrine, pseudoephedrine, and minus isoform of meth, which are commonly used as a bronchodilator or nasal decongestant in over-the-counter medications, and phentermine, which is approved as an appetite suppressant to help release weight, are potential candidates for a screen for anti-influenza agents. In addition, targeting the cellular compartments rather than the viral components could potentially reduce the frequency of drug resistance; and finding new uses for old drugs may shorten the duration to fulfill the regulatory requirements before clinical use initiated. Of note, although meth can effectively attenuate influenza A virus replication, it might not be a promising choice to use meth as an anti-influenza agent in the drug abusing population, since subjects in this particular population are more likely to be positive for HIV and/or HCV, and meth can enhance infection of these two pathogens.

Although this study was designed to investigate meth’s effect on influenza A virus replication, the concept of this study could also be applied to other illicit substances with medical uses, such as morphine (used for pain management) and cocaine (as a local anesthetic and vasoconstrictor), as well as other prescribed medicines with similar stimulant properties to meth, such as methylphenidate, which is already used for the treatment of attention deficit hyperactivity disorder (ADHD) and narcolepsy. Therefore, those extended studies could potentially provide clinical benefits to a broader population.

### Conclusions

In conclusion, the present study provides the first demonstration that meth inhibits influenza A virus replication *in vitro*, primarily via acting at the viral replication stage. Given meth’s action on attenuating influenza infections, further studies to screen other structurally similar compounds for use as an antiviral agent(s) against influenza virus infections should be pursued.
